# Bottom-up and top-down controls on *Alteromonas macleodii* lead to different dissolved organic matter compositions

**DOI:** 10.1093/ismeco/ycae010

**Published:** 2024-01-23

**Authors:** Qi Chen, Christian Lønborg, Feng Chen, Rui Zhang, Ruanhong Cai, Yunyun Li, Chen He, Quan Shi, Nianzhi Jiao, Qiang Zheng

**Affiliations:** State Key Laboratory for Marine Environmental Science, Institute of Marine Microbes and Ecospheres, College of Ocean and Earth Sciences, Xiamen University, Xiamen, 361102, China; Fujian Key Laboratory of Marine Carbon Sequestration, Xiamen University, Xiamen, 361102, China; Section for Marine Diversity and Experimental Ecology, Department of Ecoscience, Aarhus University, 4000 Roskilde, Denmark; Institute of Marine and Environmental Technology, University of Maryland Center for Environmental Science, Baltimore, MD 21202, United States; State Key Laboratory for Marine Environmental Science, Institute of Marine Microbes and Ecospheres, College of Ocean and Earth Sciences, Xiamen University, Xiamen, 361102, China; Fujian Key Laboratory of Marine Carbon Sequestration, Xiamen University, Xiamen, 361102, China; State Key Laboratory for Marine Environmental Science, Institute of Marine Microbes and Ecospheres, College of Ocean and Earth Sciences, Xiamen University, Xiamen, 361102, China; Fujian Key Laboratory of Marine Carbon Sequestration, Xiamen University, Xiamen, 361102, China; State Key Laboratory of Heavy Oil Processing, China University of Petroleum, Beijing, 102249, China; State Key Laboratory of Heavy Oil Processing, China University of Petroleum, Beijing, 102249, China; State Key Laboratory of Heavy Oil Processing, China University of Petroleum, Beijing, 102249, China; State Key Laboratory for Marine Environmental Science, Institute of Marine Microbes and Ecospheres, College of Ocean and Earth Sciences, Xiamen University, Xiamen, 361102, China; Fujian Key Laboratory of Marine Carbon Sequestration, Xiamen University, Xiamen, 361102, China; State Key Laboratory for Marine Environmental Science, Institute of Marine Microbes and Ecospheres, College of Ocean and Earth Sciences, Xiamen University, Xiamen, 361102, China; Fujian Key Laboratory of Marine Carbon Sequestration, Xiamen University, Xiamen, 361102, China

**Keywords:** dissolved organic matter, virus, DOM chemical composition, Fourier transform ion cyclotron resonance mass spectrometer (FT-ICR MS), Alteromonas macleodii

## Abstract

The effects of both bottom-up (e.g. substrate) and top-down (e.g. viral lysis) controls on the molecular composition of dissolved organic matter have not been investigated. In this study, we investigated the dissolved organic matter composition of the model bacterium *Alteromonas macleodii* ATCC 27126 growing on different substrates (glucose, laminarin, extracts from a *Synechococcus* culture, oligotrophic seawater, and eutrophic seawater), and infected with a lytic phage. The ultra-high resolution mass spectrometry analysis showed that when growing on different substrates *Alteromonas macleodii* preferred to use reduced, saturated nitrogen-containing molecules (i.e. O4 formula species) and released or preserved oxidized, unsaturated sulfur-containing molecules (i.e. O7 formula species). However, when infected with the lytic phage, *Alteromonas macleodii* produced organic molecules with higher hydrogen saturation, and more nitrogen- or sulfur-containing molecules. Our results demonstrate that bottom-up (i.e. varying substrates) and top-down (i.e. viral lysis) controls leave different molecular fingerprints in the produced dissolved organic matter.

## Introduction

Heterotrophic bacteria play pivotal roles in the marine microbial food web and biogeochemical cycles largely because of their high abundance, diversity, and metabolic activity [1]. Bacterial growth and mortality are primarily linked to the utilization of bioavailable dissolved organic matter (DOM) and the production of partly more recalcitrant DOM, respectively [2–4]. Multiple bottom-up (e.g. substrates) and top-down (e.g. viruses) factors have been demonstrated to impact the growth and mortality of microorganisms, thus playing a crucial role in shaping both the microbial community structure [5–7] and the production of DOM [8]. For example, specific bacterial groups (Gamma- and Alpha-proteobacteria) thrive under nutrient-deplete conditions [9], and different bacterial strains have been shown to produce distinct fluorescent DOM when growing on the same substrate [10, 11]. These bacterial produced DOM could be furthermore transformed by diverse microbial populations, ultimately resulting in the accumulation of recalcitrant DOM in the ocean, a process commonly referred to as the microbial carbon pump [2]. On the other hand, top-down controls (e.g. viral lysis and protistan grazing) can also influence bacterial community composition and ocean biogeochemistry [4, 12–14]. Viral lysis has been shown to daily remove up to 40% of the bacterial biomass and decrease heterotrophic production by up to 80%, releasing essential nutrients for marine microbes [13–15]. Alternatively, viral activities can regulate host physiology (e.g. transparent exopolymeric polysaccharide production) and enhance carbon sink from surface to deep waters, greatly impact ocean biogeochemistry [16, 17].

In the marine environment, a multiple of bottom-up and top-down controls exert simultaneously influences on the bacterial community, exhibiting spatial and temporal variations [18–20]. Previous studies have mainly focused on the response of bacterial abundance (BA), functions, and diversity under different bottom-up and top-down controls, while the impacts on molecular composition of the produced DOM remain to be studied. Recent works have compared the bacterial exometabolome [21] and demonstrated how different simple substrates stimulate the bacterial growth that not only leads to the production of thousands of new molecules that are similar to environmental DOM signatures [22–24] but also unique compounds [25]. On the other hand, viral lysis of phytoplankton cultures and bacteria has been shown to produce labile DOM (e.g. nitrogen/sulfur-containing molecules), which easily can be degraded by bacteria [8, 26–28], but detecting the viral molecular fingerprints might be difficult in natural samples where multiple sources and sinks work at the same time resulting in a highly complex pool [29–31]. Therefore, as both substrate and viruses contribute to the DOM pool, it is important to investigate if they have different molecular fingerprints.

The bacterial strain, *Alteromonas macleodii* is widely distributed from the surface to the deep layers in both temperate and tropical marine waters showing distinct metabolic capabilities [32, 33]. Despite the opportunistic role in response to phytoplankton blooms [34], *Alteromonas* has also been found to interact with other microbes when degrading organic matter [35, 36]. Recent studies have shown how the widely distributed *Alteromonas* strains can degrade diverse and considerable amounts of organic matter [37, 38] and be lysed by different types of viruses [39]. Because of its environmental relevance we in this study used *A. macleodii* as a model bacterium to (i) understand how bottom-up (i.e. substrate complexity) and top-down (i.e. viral lysis) controls affect the molecular characteristics of the produced organic matter and (ii) determine if we can detect a unique molecular fingerprint of viral lysis in the produced DOM.

## Materials and methods

### Experimental setup

To compare the influence of bottom-up (different substrates) and top-down (viral lysis) controls on bacterial produced DOM, we grew type strain *A. macleodii* ATCC 27126 (GenBank: GCA_000172635.2) under a range of conditions (see Fig. S1 in supporting information for details). All glassware was acid washed, rinsed with Milli-Q water, and pre-combusted (450°C, 6 h) before use. *Alteromonas macleodii* was inoculated in glucose (Sigma Aldrich) medium and cell pellets were washed three times with 0.22-μm prefiltered artificial seawater as bacterial inoculum. For bottom-up control experiment, we added five substrates: glucose, laminarin (Sigma Aldrich), three types of extracted DOM from a *Synechococcus* culture (*Syn*-DOM), oligotrophic (Oligo-DOM), and eutrophic seawater (Eu-DOM), whereas a control was prepared without any organic substrate addition. The inoculum (~2.6 × 10^5^ cells/mL) was added and triplicate 1-L glass bottles for each treatment were used and incubated in the dark at 26°C. In addition to viral lysis, bacterial derived DOM could be released by exudation and mechanical cell lysis [40]. For top-down control experiment, the bacterial inoculations were conducted in six 100-mL glass bottles amended with the glucose medium as described above. Three bottles were centrifuged to prepare exudate DOM (supernatants), and cells were mechanically lysed for mechanical lysate DOM preparation, and the other three bottles were inoculated with *Alteromonas* phage to prepare viral lysis derived DOM (for details of the two experimental setups see supporting information). All samples were filtered through pre-combusted GF 75 filters (nominal pore size 0.3 μm, Advantec) and then extracted as exudate, mechanical, and viral lysate DOM, respectively.

### BA measurement

BA was measured by staining samples with the nucleic acid-specific dye SYBR Green I (Invitrogen) [41] for 15 min in the dark and analyzed using a flow cytometry (BD Accuri C6). The cell counts were measured based on the targeted detector (FL1, 530 ± 30 nm) and two additional detectors, next to forward (FSC) and side (SSC) scatter.

### DOM analysis

The dissolved organic carbon (DOC) concentration, fluorescent and molecular DOM analysis, and data processing have been previously described (for details see the supporting information) [23]. The mass measurement of Day 0 for control was also used for glucose and laminarin as polar glucose and laminarin cannot be extracted. After processing the average intensity-weighted of different elementary formulas (CHO, CHON, CHOS, and CHONS), ratios of hydrogen to carbon (H/C) and oxygen to carbon (O/C) were calculated for each sample [42]. Double bond equivalents (DBE), which encompass the sum of unsaturations and rings, along with normalized unsaturation (DBE/C) and the modified aromaticity index (AI_mod_), reflecting the extent of aromatic and condensed compounds, were calculated as previously described [43, 44].

### Statistical analysis

All statistical analyses were performed in R 3.6.1 (www.R-project.org). Correlations were performed using Pearson coefficients, and visualized with the “corrplot” package with a significance level of *P* < .05. Principal component analysis (PCA) was used to investigate the compositional differences of the organic molecular characteristics produced by *A. macleodii* when processing different substrates. Analysis of similarities (ANOSIM) was conducted to compare significant differences in the molecular characteristics in different substrates. Both PCA and ANOSIM analyses were performed using the package “vegan.” The significance of molecular composition and characteristics between the two groups data comparison was tested using Wilcoxon tests and Student’s *t*-test, respectively. We applied inter-sample rankings analysis that ranked individual molecular formulas based on relative intensity in each sample and then compared the ranks of common formulas across samples to distinguish dominant formulas at different time-points/treatments [45, 46]. Spearman’s correlation was conducted between four fluorescent components and identified molecular formulas at a 95% confidence limit, as described by Stubbins *et al*. [47].

## Results and discussion

Here, we investigated the molecular signatures and elemental compositions of the DOM that were produced under different substrates, and induced by viral lysis of *A. macleodii*. In this study, we compared characteristics of *A. macleodii* growing on glucose, laminarin, and *Syn*-DOM [23] together with the Oligo-DOM and Eu-DOM treatments. The DOM produced by *A. macleodii* because of mechanical lysing and viral lysis in the glucose treatment are also shown for the first time.

### Bacterial growth with different organic substrates

**Figure 1:**
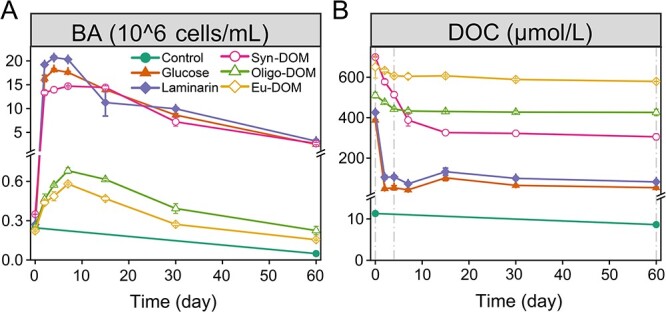
Changes in the BA (A) and DOC concentrations (B) in the *Alteromonas* culture incubations in the control, glucose, laminarin, DOM extracted from a *Synechococcus* culture (*Syn*-DOM), oligotrophic (Oligo-DOM), and eutrophic seawater (Eu-DOM) treatments, respectively. Dash line (Days 0, 4, and 60) represents sampling times for mass measurement.

BA showed the typical logistic growth curves in all substrate-amended treatments, except the slight decline in the control (Fig. 1A). Initial exponential growth varied depending on the substrates, and BA increased rapidly from initially ~2.6 ± 0.5 × 10^5^ to ~1.7 ± 0.3 × 10^7^ cells/mL within the first 4 days in the glucose, laminarin and *Syn*-DOM treatments. While BA peaked (~ 6.3 ± 0.6 × 10^5^ cells/mL) at day seven in the Oligo-DOM and Eu-DOM treatments. Bacterial growths mirrored the DOC consumption with the highest consumption in the glucose (86%) and laminarin (81%) treatments, followed by the *Syn*-DOM (56%), Oligo-DOM (17%), and lastly, the Eu-DOM (11%) treatment (Fig. 1B). Despite the role of substrate, the concentrations of DOM and inorganic nutrient also have a role in regulating bacterial growth as well as their products [48, 49]. Our results imply that the substrate source/composition was a major factor controlling the growth of *A. macleodii*, which is in accordance with recent studies showing that source properties determine microbial degradation [50, 51].

### Fluorescent DOM utilization and production of varying substrates

The parallel factor analysis [52] resolved four fluorescent components (Fig. S2A). It is noteworthy that we compared the relative change of the fluorescence intensity among frozen samples that may not provide quantitative comparison to optical DOM properties in natural seawater [53, 54]. Components C1 (Ex/Em wavelengths: 270/308 nm) and C2 (Ex/Em wavelengths: 275/337 nm) are commonly recognized as tyrosine-like and tryptophan-like components, respectively [55, 56]. The C1 and C2 levels normalized to DOC concentration increased with increasing BA (Fig. S2C, *r* = 0.46, *P* < .01 and *r* = 0.50, *P* < .01, respectively), suggesting that these two protein-like components are associated with bacterial growth and metabolic activities. These protein-like components contain a fraction of the labile DOM [56, 57], and *Alteromonas* is able to produce these two protein-like components even when degrading simple compounds such as glucose [58]. Component C3 (Ex/Em wavelengths 310/392 nm) and component C4 (Ex/Em wavelengths 365/459 nm) were similar to fluorescent humic-like components [59, 60]. Indeed, C3 normalized to DOC concentration generally increased over time in all incubations (*r* = 0.71, *P* < .01), implying a production of relative recalcitrant components [61]. Component C4 was relatively stable in each treatment, suggesting a recalcitrant nature.

### Substrate impacts on the microbial transformation of organic matter

A PCA was conducted to investigate the molecular compositional differences of the DOM produced by *A. macleodii* when processing different substrates (Fig. S3). The molecular characteristics significantly differ among different treatments (ANOSIM, *r* = 0.79, *P* = .001) rather than incubation time (ANOSIM, *P* > .05). The different characteristics of molecules could be driven by the source of the substrate (naturally enriched DOM versus simple compounds). We conducted correlation analysis to discern the similarity in behaviors of fluorescent and molecular DOM (Fig. S4). The protein-like components C1 and C2 were positively correlated with formulas showing lower average O/C (0.22) and higher average H/C (1.65) values, respectively. These formulas generally presented labile characteristics, aligning with their microbial bioavailability [62, 63]. On the contrary, humic-like components C3 and C4 were positively correlated with higher average O/C (0.44 and 0.46) formulas. Both humic-like component and oxidized molecules could be formed as a result of bacterial transformation of labile organic substrates [64, 65].

**Figure 2:**
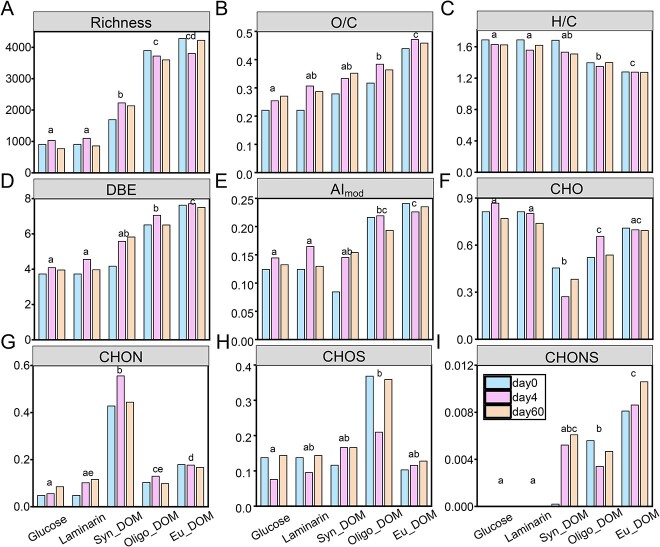
Boxplots (median, min, and max) of molecular characteristics (richness (number of identified formulas, A), average intensity-weighted of ratios of O/C (B), H/C (C), DBE (D), modified aromaticity index (AI_mod_, E), and different elementary formulas (CHO, CHON, CHOS, and CHONS, F–I) of the identified DOM in the *Alteromonas* cultures growing on glucose, laminarin, and DOM extracted from a *Synechococcus* culture (*Syn*-DOM), oligotrophic (Oligo-DOM), and eutrophic seawater (Eu-DOM) during the incubation (including initial, intermediate, and final timepoint). The different letters differ significantly from each treatment, *P* < .05.

Generally molecular richness (number of identified formulas), average intensity-weighted of the O/C ratio, DBE and AI_mod_ values increased (Fig. 2A–D), whereas the H/C ratio declined (Fig. 2E) from simple compounds (glucose and laminarin) to complex DOM (*Syn*-DOM, Oligo-DOM, and Eu-DOM) treatments. Consistent with the decrease in DOC consumption observed from simple to complex substrates, these finding confirm labile signature of hydrogen saturated compounds [62] and imply a lower bioavailability of oxidized, unsaturated, and aromatic substrate sources. Although molecular characteristics remained largely consistent after incubation for each treatment, an inter-sample ranking analysis was further applied to compare the common formulas (encompassed over 80% relative intensity of identified formulas) among different sampling times to resolve the molecular transformation signature. The dominant formulas (top rank) were classified as preformed formulas (dominating on Day 0), intermediated formulas (dominating on Day 4), and remaining formulas (dominated on Day 60) in each substrate treatment (Fig. S5A). It is noted that a formula can be assigned as more than one type in different treatments so that the unique preformed, intermediated, and remaining formulas across five treatments were selected for further comparison. Overall, the unique remaining formulas showed significantly lower H/C ratios but higher O/C ratios and DBE/C values (Wilcoxon test, *P* < .01) compared with the preformed and intermediated formulas (Fig. S5B). This result demonstrated that *A. macleodii* preferentially use reduced, saturated organic molecules while they produce or are unable to degrade unsaturated molecules. Our findings are well in line with previous studies showing that microbial degradation of organic matter result in the production of more oxidized formulas [62, 66, 67]. Although recent study has reported that *Alteromonas* cultures can also produce reduced compounds when growing on different polysaccharides [25].

**Figure 3:**
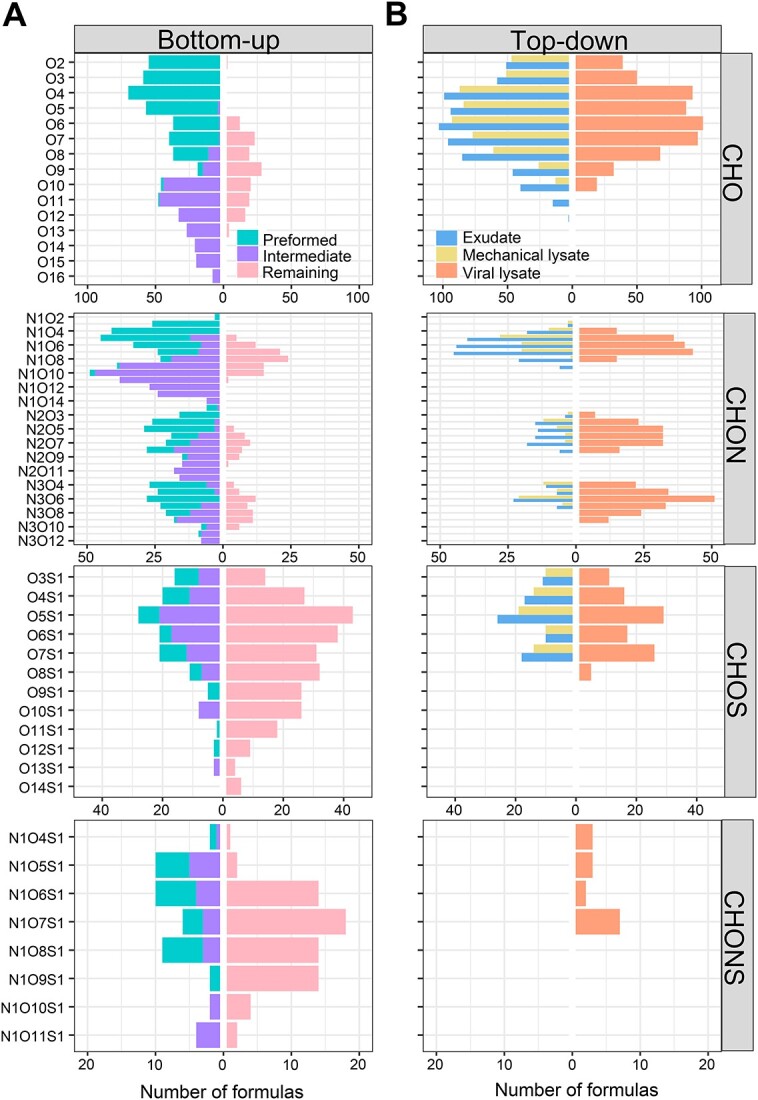
Number of different formula species (Ox, NyOx, NyOxSz, and OxSz) of elementary formulas (CHO, CHON, CHOS, and CHONS, respectively) in unique preformed (dominating on Day 0), intermediated (dominating on Day 4), and remaining (dominating on Day 60) formulas in the *Alteromonas* incubations growing on different substrates (A), and exudate, mechanical and viral lysate formulas in the *Alteromonas* incubations growing on glucose (B).

In general, there were more CHO and CHON containing formulas in the bioavailable (preformed and intermediated) formulas (in total 577 and 753, respectively) than the remaining formulas (149 and 193) (Fig. 3A). In contrast, the numbers of CHONS and CHOS containing formulas were more abundant in the remaining formulas (69 and 274) compared with the bioavailable formulas (16 and 24). Additionally, O4 formula species were dominated in preformed (165), in contrast to intermediated (21) and remaining (35) formulas (Fig. 3A). O10 formula species were mostly found in intermediated (122), as opposed to preformed (6) and remaining (4) formulas. The remaining formulas (112) contained a greater number of O7 formula species compared with preformed (88) and intermediated (47) formulas. Commonly, heterogeneous molecules containing N or S are found in phytoplankton produced DOM [62, 68], and these are particularly enriched in viral lysates of *Synechococcus* [26, 28], which are also highly bioavailable. Meanwhile, our results showed the bioavailable fraction of CHO containing formulas as previously found in river or wastewater [69, 70]. Overall, these findings suggest that substrate source is important in determining the proportion of molecular formulas that can be degraded by microbes. However, it should be noted that in this study we used a single *Alteromonas* culture and the findings might be different if natural microbial populations were used.

### Viral impacts on the chemical composition of *Alteromonas*-produced organic matter

The inter-sample ranking analysis showed around 90% (relative intensity) of the identified common formulas among the directly exudate, mechanical lysate, and viral lysate DOM produced by *A. macleodii* (Fig. S6A). Compared with directly exudate DOM, the mechanical and viral lysate had significantly (Wilcoxon test, *P* < .01) higher H/C ratios but lower DBE/C values (Fig. S6B), suggesting that the lysed material consisted of more high hydrogen saturated organic compounds. These results are opposed to recent studies in which viral lysis of a bacterial culture (*Rhodobacteraceae*) consisted of more aromatic and unsaturated formulas with higher O/C values [31], and *Synechococcus* viral lysates contained mainly unsaturated and oxygen-rich formulas [29]. Because different microbial groups were used in our and the previous studies, viral lysis of different groups will lead to the differences in the DOM composition. In addition, our results also demonstrate that different substrate types affected the bacterial growth, which may again influence the amount and chemical composition of the resulting viral lysate [71]. Further studies are therefore needed to determine if the complex interactions between different substrates and viral lysis influence the chemical composition of the resulting DOM.

Interestingly, the viral lysate comprised unique molecules (e.g. N2O, N3O, and NOS species), which were not found in the cell exudate and mechanically lysed DOM (Fig. 3B). These unique virus-mediated formulas had generally low O/C and DBE/C ratios but high H/C ratios, suggesting that viral infection could provide novel reduced heterogeneous molecular formulas with high hydrogen saturation (Fig. S6B). Previous studies have reported that viral lysis of cyanobacteria can release peptides (e.g. phycoerythrin), and a series of N-containing compounds (amino acids/oligopeptides, nucleosides/nucleotides, and biogenic amines), suggesting that viral lysis could contribute to the dissolved organic nitrogen pool [28, 29, 72]. Viral-induced bacterial lysis can also result in the release of amino acids [71] and ammonium [73] driving nitrogen cycling. Similarly, we demonstrate that the viral lysate from *A. macleodii* is enriched in N-containing saturated molecules. Although viruses themselves are enriched in N and P and they thereby can enrich the N- and P-containing formulas in the resulting DOM [31, 74], more importantly, viruses are able to impact host metabolisms by changing substrate utilization and energy production in order to produce progeny [75]. Such virus-mediated changes have been shown to alter the host cellular metabolic state [76]. These viral infection processes could change intracellular organic composition (e.g. fatty acid and pigment) [77, 78] and/or create unique extracellular metabolic products (e.g. halogenation) [30].

### Implications

**Figure 4:**
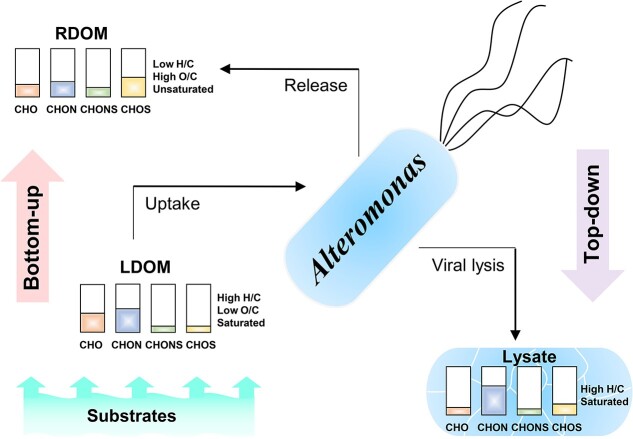
The diagram of DOM transformations (molecular characteristics and elementary compositions (CHO, CHON, CHOS, and CHONS, respectively)) of the *Alteromonas* culture under the bottom-up (i.e. substrate compositions) and top-down (i.e. viral lysis) controls. LDOM and RDOM represent relative labile and recalcitrant DOMs, respectively, for the *Alteromonas* culture.

In this study, we demonstrate how bottom-up (i.e. substrate types) and top-down (i.e. viral lysis) controls can influence the molecular composition of DOM produced by *A. macleodii* (Fig. 4). The *A. macleodii* culture without the addition of viruses produced oxidized, unsaturated molecules with higher aromaticity. With the addition of the lytic phage, *A. macleodii* produced DOM compounds containing more hydrogen saturated and novel heterogeneous molecular formulas.

In the ocean, bacteria transform cell-excreted DOM and viral lysis-released DOM, resulting in the production of refractory DOM with long turnover times [2, 27]. We demonstrate that viral lysates had similar composition (i.e. N-riched with more hydrogen saturated) compared with the compounds that are preferential degraded by *A. macleodii* implying the potential labile nature of these viral lysates. However, the degradation of the viral lysate and the potential contribution of the recalcitrant DOM pool require further investigation. This study considers two DOM sources (top-down and bottom-up) and shows the different impacts of these two major organic matter sources on microbial transformation and resulting DOM composition.

## Funding

National Natural Science Foundation of China (42188102, 42222604, 92351303, 92251306, 41861144018); the Chinese Academy of Sciences (project XK2022DXA001); Marine Economic Development Program of Fujian Province (Grant No. FJHJF-L-2022-11); the Fundamental Research Funds for the Central Universities (20720190095); funds from the Third Institute of Oceanography, Ministry of Natural Resources (grant EPR2022001); the Independent Research Fund Denmark Grant (1127-00033B).

## Conflicts of interest

The authors declare no competing interests.

## Data availability

FT-ICR MS data are available at https://doi.org/10.6084/m9.figshare.21779609.

## Supplementary Material

Supporting_information_ISMEC_ycae010
