# Supplementary material for: Bottom-up and top-down controls on Alteromonas macleodii lead to different dissolved organic matter compositions
Source: ISME Commun. 2024 Jan 23;4(1):ycae010. doi: 10.1093/ismeco/ycae010 (PMC10926778; doi:10.1093/ismeco/ycae010)
Supplement: Supporting_information_ISMEC_ycae010 [file supporting_information_ismec_ycae010.docx]

**Supporting Information for**

**Bottom-up and top-down controls on *Alteromonas macleodii* lead to different dissolved organic matter compositions**

Qi Chen ^1,2^, Christian Lønborg ^3^, Feng Chen^4^, Rui Zhang ^1,2^, Ruanhong Cai ^1,2^, Yunyun Li ^5^,

Chen He ^5^, Quan Shi ^5^, Nianzhi Jiao ^1,2*^, Qiang Zheng ^1,2*^

^1^State Key Laboratory for Marine Environmental Science, Institute of Marine Microbes and Ecospheres, College of Ocean and Earth Sciences, Xiamen University, Xiamen, China

^2^Fujian Key Laboratory of Marine Carbon Sequestration, Xiamen University, Xiamen, China

^3^Section for Marine Diversity and Experimental Ecology, Department of Ecoscience, Aarhus University, Roskilde, Denmark

^4^Institute of Marine and Environmental Technology, University of Maryland Center for Environmental Science, Baltimore, Maryland, United States

^5^State Key Laboratory of Heavy Oil Processing, China University of Petroleum, Beijing, China

**Address correspondence to Qiang Zheng,** [zhengqiang@xmu.edu.cn](mailto:zhengqiang@xmu.edu.cn)

**Nianzhi Jiao,** [jiao@xmu.edu.cn](mailto:jiao@xmu.edu.cn)

**Figure S1**. Overview of the experimental design used to test the impact of bottom-up (i.e. substrates) and top-down (i.e. virus) controls on *Alteromonas* culture. We here used artificial seawater (ASW) as control and with the addition of different substrates (glucose, laminarin, extracted DOM of a *Synechococcus* culture (*Syn*-DOM), oligotrophic (Oligo-DOM) and eutrophic seawater (Eu-DOM).


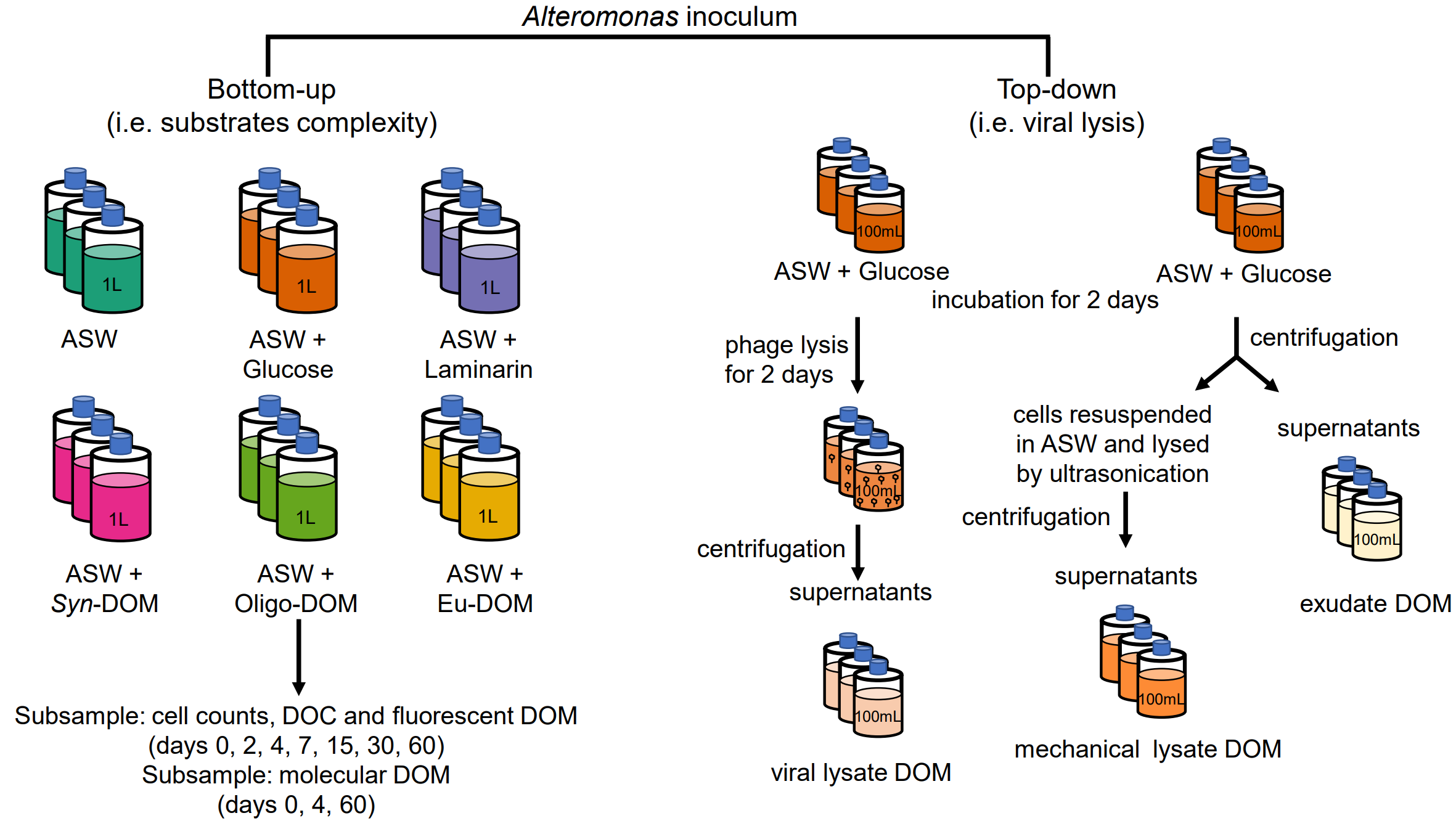


**Figure S2**. Four fluorescent component (A) and changes in fluorescent component intensity (B) during the incubations, and correlations between bacterial abundances, incubation time concentrations and fluorescent intensity normalized to dissolved organic carbon (DOC) concentration (C), significance levels (*p* < 0.05) were shown.


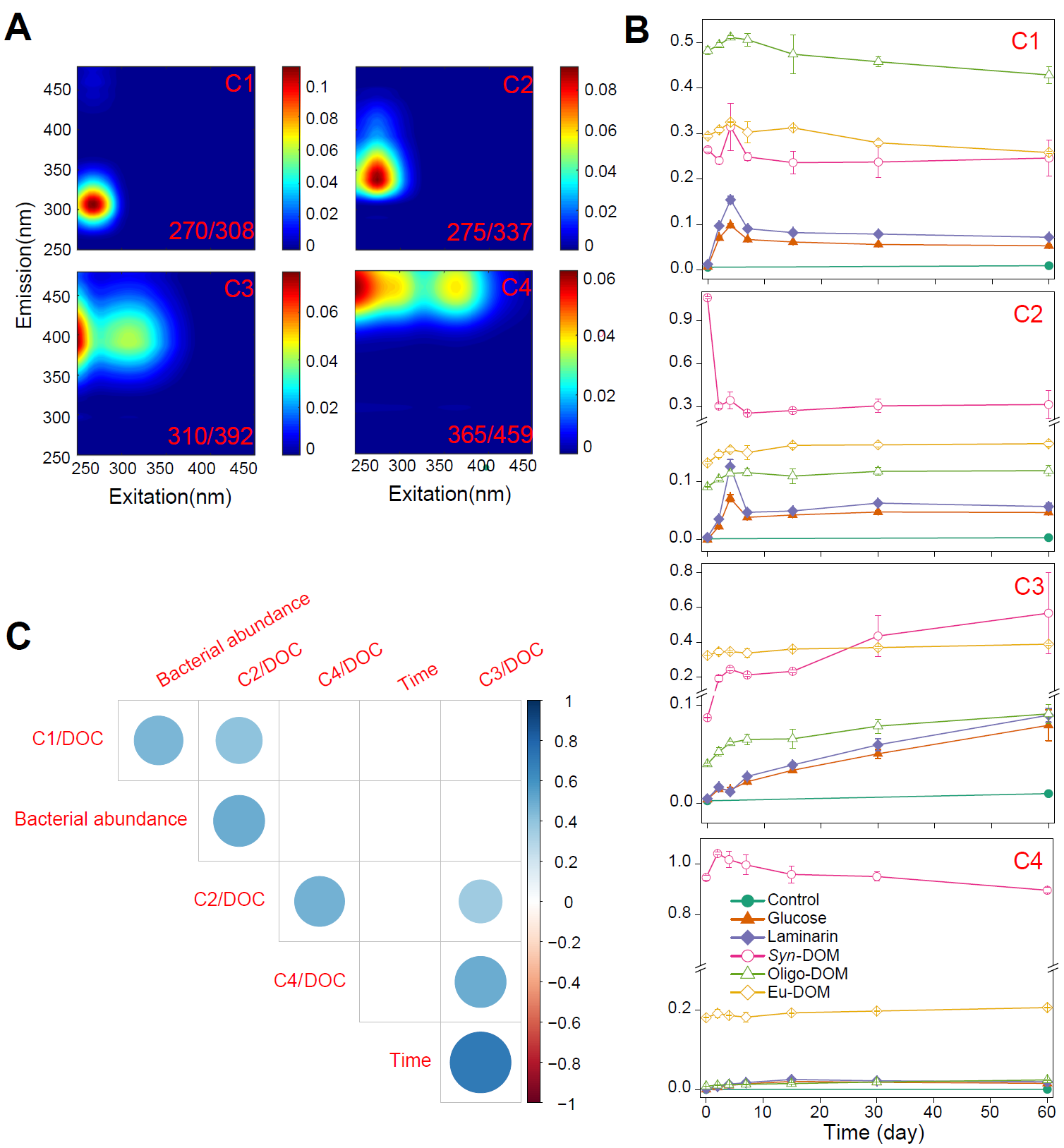


**Figure S3**. Principal component analysis (PCA) plots derived from molecular characteristics (richness (number of identified formulas), average intensity-weighted of the ratios of hydrogen to carbon (H/C) and oxygen to carbon (O/C), the double bond equivalents (DBE), modified aromaticity index (AI_mod_) and different elementary formulas (CHO, CHON, CHOS, and CHONS)) of the identified dissolved organic matter (DOM) in the *Alteromonas* culture experiments with glucose, laminarin, extracted DOM of a *Synechococcus* culture (*Syn*-DOM), oligotrophic (Oligo-DOM) seawater and eutrophic seawater (Eu-DOM).


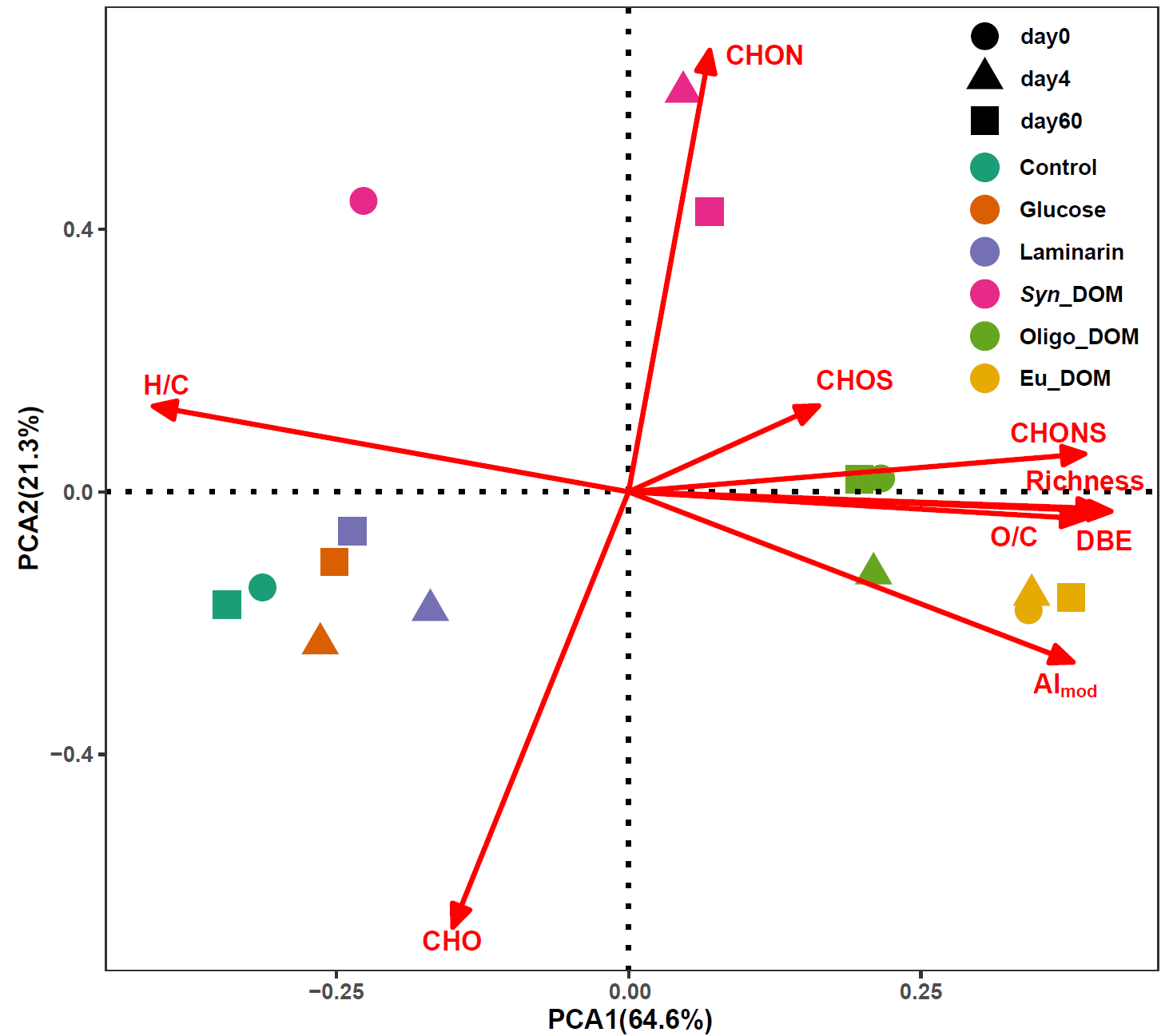


**Figure S4.** Inter-sample rankings analysis (Herzsprung et al., 2012, 2017) of common formulas (percentage represents relative intensity) in the *Alteromonas* culture experiments with different substrates (glucose, laminarin, extracted DOM of a *Synechococcus* culture (*Syn*-DOM), oligotrophic (Oligo-DOM) and eutrophic seawater (Eu-DOM) at different time-points (A). Colors represent ranks (top rank indicates dominant molecular formulas in each sample). Violin plots, which includes the box plot (median, min and max) showing dominant formulars (days 0, 4, 60, respectively) of different molecular characteristics (hydrogen to carbon (H/C) and oxygen to carbon (O/C) ratios as well as the normalized unsaturation (DBE/C)) in the *Alteromonas* culture with the different substrates (B). Unique category represents formulas exiting in only one formular group from all substrates. Significance levels: * for *p* < 0.05, **for *p* < 0.01, ***for *p* < 0.001 and ns for *p* > 0.05.


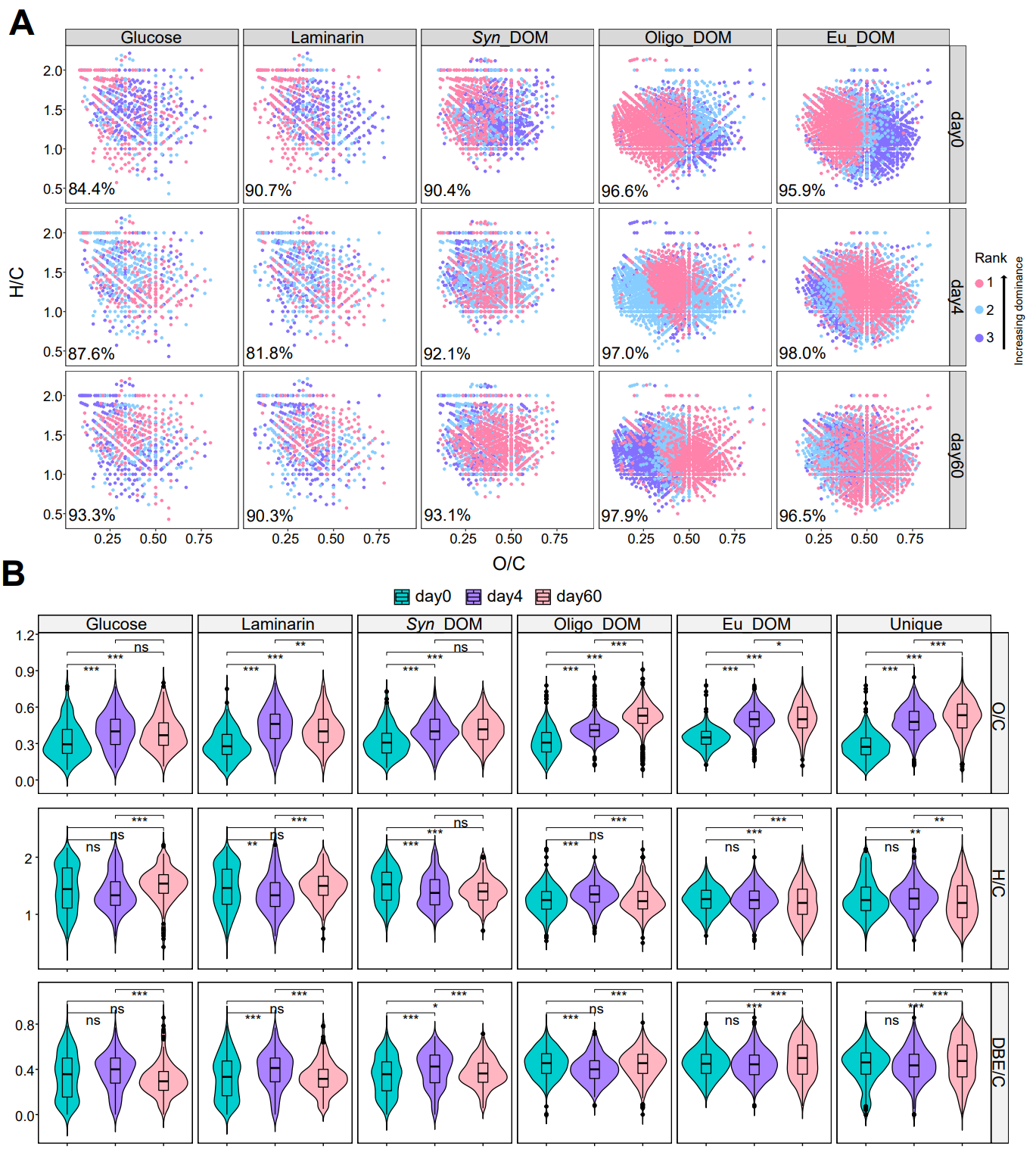


**Figure S5.** Inter-sample rankings analysis (Herzsprung et al., 2012, 2017) of common formulas (percentage represents relative intensity) from directly exudate, mechanical and viral lysate in the *Alteromonas* cultures growing on glucose (A). Colors represent ranks (top rank indicates dominant molecular formulas in each sample). Violin plots, which includes the box plot (median, min and max) showing common and unique formulas of different molecular characteristics (hydrogen to carbon (H/C), oxygen to carbon (O/C) and normalized unsaturation (DBE/C)) from exudate, mechanical lysate and viral lysate in the *Alteromonas* culture growing on glucose (B). Significance levels: * for *p* < 0.05, **for *p* < 0.01, ***for *p* < 0.001 and ns for *p* > 0.05.


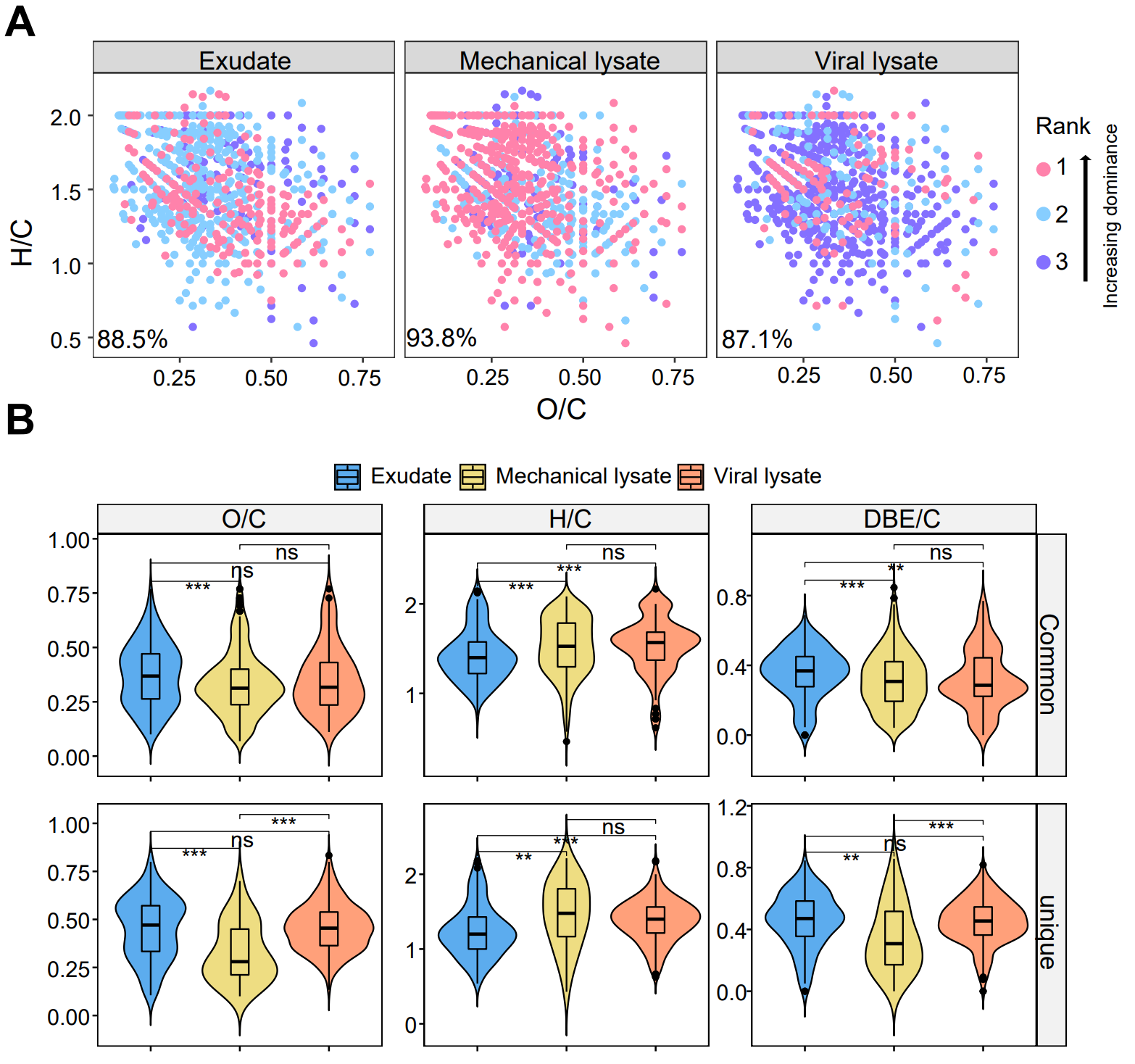


**Experimental process**

Artificial seawater (ASW) was modified from Kester et al. [1], containing NaCl (24 g L^−1^), KCl (0.67 g L^−1^), KBr (0.1 g L^−1^), NaF (0.04 g L^−1^), H_3_BO_3_ (0.03 g L^−1^), CaCl_2_ (0.05 g L^−1^), NaHCO_3_ (0.2 g L^−1^), MgSO_4_·7H_2_O (5 g L^−1^), NaNO_3_ (6 mg L^−1^), NH_4_Cl (4 mg L^−1^) and KH_2_PO_4_ (2 mg L^−1^) dissolved in Milli-Q water. The first four salts were pre-combusted (450℃, 6 h) to remove contaminating organic carbon. Five substrates (~500 µmol C L^−1^) comprising of different complexity and source (including glucose (Sigma Aldrich), laminarin (Sigma Aldrich), extracted DOM from a *Synechococcus* culture (*Syn*-DOM), oligotrophic (Oligo-DOM) and eutrophic seawater (Eu-DOM) were added to the ASW, respectively, while a control (~4 µmol C L^−1^) was prepared without any organic substrate addition. The amended substrate concentrations were comparable to dissolved organic carbon (DOC) levels found during coastal bloom periods [2, 3]. The *Syn*-DOM was extracted from cells lysate of *Synechococcus* sp. XM-24 (GenBank: GCA_003149585.1) during the exponential phase, and Oligo-DOM and Eu-DOM were concentrated with solid phase extraction from surface seawater near Yongxing Island, China (~17°N, ~112°E, DOC concentration: ~81 µmol C L^−1^) and Xiamen Island, China (~24°N, ~118°E, DOC concentration: ~126 µmol C L^−1^), respectively [4, 5]. The resulting medium solutions were filtered through a pre-washed (1 L Milli-Q water) 0.22-µm filter. Additionally, we prepared six bottles amended with bacterial inoculum (~2.6×10^5^ cells·mL^-1^) in glucose medium, and inoculated three of these with *Alteromonas* phage belonging to *Siphoviridae* isolated from an subtropical estuary (~21°N and ~113°E). The virus had been pre-activated in *Alteromonas* culture using glucose medium. Viral lysate was filtered through a pre-washed 0.22-μm ﬁlter and added in an approximate 1:1 virus to host ratio (~1.0×10^7^ cells·mL^-1^).

For the different substrate-amended experiments, to prevent microbial contamination during subsampling all bottles were handled in an ultraviolet sterilized clean bench. Each bottle was subsampled for bacterial abundance, DOC concentration and fluorescent DOM (FDOM) at seven time-points (days 0, 2, 4, 7, 15, 30, 60), while DOM chemical analysis was performed on days 0, 4 and 60. Briefly, for bacterial counts 2 mL of a sample was mixed with glutaraldehyde (1% v/v), flash frozen in liquid nitrogen and stored -80°C until analysis. For DOM analysis, 40 mL water sample were filtered through a GF 75 ﬁlter (pre-combusted, nominal pore size 0.3-μm, Advantec). Subsequently, 20 mL filtered samples were transferred directly into two 40 mL pre-combusted glass vials and stored at -20°C for DOC and FDOM measurements, respectively. For the DOM chemical analysis, 100 mL filtrate was acidified to a pH of 2 in a 250 mL glass bottle. All glassware was acid washed, rinsed with Milli-Q water, and pre-combusted before use.

To determine if viruses impacted the chemical composition of the *Alteromonas* produced DOM we collected directly exudate, mechanical and viral lysate of the *Alteromonas* cultures growing on glucose. We first harvested cells from 100 mL phage-free cultures after two days growth by centrifugation at 4000g for 10 min. The supernatants were collected as the exudated DOM filtrates. Cell pellets were following resuspended in 100 mL ASW and lysed mechanically in an ice bath for 30 min with 2 s running and 4 s intervals using a 650-W sonicator (SM-650D; Shunma Tech., Nanjing, China). The lysed cell suspensions were again centrifuged, and the supernatants were collected to represent the mechanical lysate DOM. The viral infected cultures were kept for further two days, where after these cultures were centrifuged and the supernatants were collected as the viral lysate DOM. All samples were filtered through pre-combusted GF 75 ﬁlters and then extracted to prepare exudate, mechanical and viral lysate DOM of the *Alteromonas* cultures, respectively.

**DOM measurement and analysis**

The DOC concentrations were measured by high-temperature (680°C) catalytic oxidation with a Shimadzu TOC-VCPH analyzer. Samples were defrosted and acidiﬁed to a pH of 2 with 85~90% phosphoric acid. Concentrations were determined by subtracting a Milli-Q water blank and dividing by the slope of a standard curve made from potassium hydrogen phthalate. The consistency between runs was verified by comparing stabilized measurement of “standard solutions” (0.2-µm filtered coastal seawater, 97.0 ± 1.0 μmol/L). The degraded (“bioavailable”) DOC was in this study defined as the difference between the initial and final DOC concentrations.

Excitation (Ex)-emission (Em) matrices (EEMs) were measured using a Horiba Aqualog. Defrosted samples (room temperature) were scanned from 240–600 nm (every 5 nm) using a 1-cm path length quartz cuvette. Emission scans ranged from 248 to 829 nm (every 2.33 nm) at a 2 s integration time. Parallel factor analysis (PARAFAC) was applied to calculate individual fluorescent DOM component in Matlab 2018 [6]. Fluorescence intensity in the EEMs were normalized to the Raman peak and reported as Raman units (RU) (excitation at 350 nm, emission from 371–428 nm) [7].

Solid phase extraction for DOM chemical characterization was performed following previous published procedures [8]. Briefly, 100 mL of filtrate was acidified (pH 2) with formic acid (CNW), and extracted using cartridges (200 mg, pre-activated with methanol (Sigma Aldrich) and 0.01 M formic acid, Agilent Bond Elut PPL, USA). After extraction, cartridges were rinsed with 6 mL of 0.01 M formic acid to remove residual salts, and then eluted with 2 mL methanol for mass analysis. Fourier transform ion cyclotron resonance mass spectrometer (FT-ICR MS) was performed using a 9.4 T Bruker Apex Ultra with an Apollo II electrospray ion source operated in negative mode [9]. The operating settings for the negative-ion ESI analysis include spray shield voltage (4.0 kV), capillary column introduced voltage (4.5 kV), and capillary column end voltage (320 V). Ions accumulated in the collision cell for 0.2 s before being transferred into the ICR cell with a time-of-flight of 1.1 ms. The detected mass range is between 200–800 Da. Samples dissolved in methanol were injected into the electrospray source at an infusion flow rate of 250 μL/h and 128 single scans were added for each mass spectrum. Subsequently, a high-quality mass peak (*m/z* 371.0620 corresponding to [C_15_H_16_O_11_–H]^−^) at the center of the mass range was selected as a reference peak. The resolution power of this peak was higher than 280,000. Two sets of mass peaks near the most abundant ones in the spectrum were selected to evaluate the relative abundance distribution. For calibration, the mass spectrometer was initially calibrated with sodium formate and recalibrated with a known mass series of the Suwannee River fulvic acids (SRFA), which provides a mass accuracy of 0.2 ppm or higher for the mass range of interest. The mass peaks with signal-to-noise (s/n) ratio greater than 4 were exported for the formula assignment, and s/n ratio greater than 6 were selected for further analysis. All elemental assignment follows: (1) the number of H atoms should be at least 1/3 that of C atoms and less than that of 2C + N + 2; (2) the sum number of N and H atoms should be even; and (3) the H/C and O/C value should be less than 3 and 1.5, respectively [10]. Matched formulas consist of the elemental combinations of ^12^C_1−60_, ^1^H_1−120_, ^14^N_0−3_, ^16^O_0−30_, and ^32^S_0−1_. Only peaks detected in at least two biological replicates were considered, relative intensity of formulas were presented as averages of replicates.

**Reference**

1. Kester D R, Duedall I W, Connors D N, et al. Preparation of Artificial Seawater1. Limnol. Oceanogr. 1967; 12: 176-179.

2. Billen G, Fontigny A. Dynamics of a Phaeocystis-dominated spring bloom in Belgian coastal waters. II. Bacterioplankton dynamics. Mar. Ecol. Prog. Ser. 1987; 37: 249-257.

3. Oh Y H, Lee Y W, Kim T H. In situ production of dissolved organic carbon (DOC) by phytoplankton blooms (Cochlodinium polykrikoides) in the southern sea of Korea. J. Sea Res. 2018; 138: 19-23.

4. Chen Q, Chen F, Gonsior M, et al. Correspondence between DOM molecules and microbial community in a subtropical coastal estuary on a spatiotemporal scale. Environ. Int. 2021; 154: 106558.

5. Zheng Q, Chen Q, Cai R, et al. Molecular characteristics of microbially mediated transformations of Synechococcus-derived dissolved organic matter as revealed by incubation experiments. Environ. Microbiol. 2019; 21: 2533-2543.

6. Murphy K R, Stedmon C A, Graeber D, et al. Fluorescence spectroscopy and multi-way techniques. PARAFAC. Anal. Methods. 2013; 5: 6557-6566.

7. Lawaetz A J, Stedmon C A. Fluorescence intensity calibration using the Raman scatter peak of water. Appl. Spectrosc. 2009; 63: 936-940.

8. Dittmar T, Koch B, Hertkorn N, et al. A simple and efficient method for the solid-phase extraction of dissolved organic matter (SPE-DOM) from seawater. Limnol. Oceanogr. Meth. 2008; 6: 230-235.

9. He C, Zhang Y, Li Y, et al. In-House Standard Method for Molecular Characterization of Dissolved Organic Matter by FT-ICR Mass Spectrometry. Acs Omega. 2020; 5: 11730-11736.

10. Kujawinski E B, Behn M D. Automated analysis of electrospray ionization fourier transform ion cyclotron resonance mass spectra of natural organic matter. Anal. Chem. 2006; 78: 4363-4373.
